# Stability of personality traits over a five-year period in Swedish patients with schizophrenia spectrum disorder and non-psychotic individuals: a study using the Swedish universities scales of personality

**DOI:** 10.1186/s12888-018-1617-y

**Published:** 2018-02-27

**Authors:** Tomas Fagerberg, Erik Söderman, J. Petter Gustavsson, Ingrid Agartz, Erik G. Jönsson

**Affiliations:** 1 Human Brain Informatics (HUBIN), Department of Clinical Neuroscience, Centre for Psychiatric Research, Psychiatry Section, Karolinska Institutet and Hospital, Stockholm, Sweden; 2 Division of Psychology, Department of Clinical Neuroscience, Karolinska Institutet, Stockholm, Sweden; 3 NORMENT, KG Jebsen Centre for Psychosis Research, Institute of Clinical Medicine. Psychiatry section, University of Oslo, Oslo, Norway; 4 Department of Psychiatric Research, Diakonhjemmet Hospital, Oslo, Norway

**Keywords:** Schizophrenia, Personality, Swedish universities scale of personality (SSP)

## Abstract

**Background:**

Personality is considered as an important aspect in persons with psychotic disorders. Several studies have investigated personality in schizophrenia. However, no study has investigated stability of personality traits exceeding three years in patients with schizophrenia. This study aims to investigate the stability of personality traits over a five-year period among patients with schizophrenia and non-psychotic individuals and to evaluate case-control differences.

**Methods:**

Patients with psychotic disorders (*n* = 36) and non-psychotic individuals (*n* = 76) completed Swedish universities Scales of Personality (SSP) at two occasions five years apart. SSP scores were analysed for effect of time and case-control differences by multiple analysis of covariance (MANCOVA) and within-subjects correlation.

**Results:**

MANCOVA within-subjects analysis did not show any effect of time. Thus, SSP mean scale scores did not significantly vary during the five-year interval. Within subject correlations (Spearman) ranged 0.30–0.68 and 0.54–0.75 for the different SSP scales in patients and controls, respectively. Patients scored higher than controls in SSP scales Somatic Trait Anxiety, Psychic Trait Anxiety, Stress Susceptibility, Lack of Assertiveness, Detachment, Embitterment, and Mistrust.

**Conclusion:**

The stability of the SSP personality trait was reasonably high among patients with psychotic disorder, although lower than among non-psychotic individuals, which is in accordance with previous research.

## Background

Psychotic disorders are often chronic and severe. Schizophrenia is a psychotic disorder that affects about 0.5% of the population [1] and has a lifetime prevalence of 1% [2]. Personality can affect symptoms and social functioning in schizophrenia [3]. Relationships have been observed between certain personality traits and the subsequent development of psychotic symptoms, psychosis and schizophrenia [4–6]. There is also studies reporting associations between individual differences in personality traits in patients with schizophrenia and symptom severity, occupational functioning, substance use, violent behavior, social isolation, and suicidal ideation, for review see [7]. This makes personality an important aspect of psychosis and schizophrenia. Several studies have investigated personality in schizophrenia [8–11]. However, there is a dearth of previous studies that have analysed stability of personality traits in patients with psychotic disorder. Personality is generally thought of as stable, although changes occur, especially during childhood and adolescence but also during adulthood, especially late in life [12]. To date, three studies have used various Five-Factor Model (FFM) questionnaires, namely NEO-Personality Inventory (NEO-PI), NEO-Five Factor Inventory (NEO-FFI), and NEO-Personality Inventory Revised (NEO-PI-R) and one that used the 168 item version of the Minnesota Multiphasic Personality Inventory (MMPI-168) to assess stability of personality traits in this patient group. Kentros et al. (1997), investigated 21 patients with schizophrenia and schizoaffective disorders (mean age 34 years) over a six-month period using test-retest correlations [13]. They found strong correlations for Neuroticism, Extraversion, Openness and Conscientiousness whereas for Agreeableness the strength of the correlation was moderate. The authors concluded that FFM personality traits remain stable over time, despite fluctuations in psychotic positive symptoms.

In another previous study [14], the mean-level of stability of personality traits was investigated in 79 patients with first-episode psychosis (mean age 24 years) over a three-month period. Statistical analysis using paired *t*-test showed no significant differences between baseline and follow-up, indicating that FFM personality traits remained stable over time.

Boyette et al. [15] investigated three-year temporal stability of FFM traits in 91 patients with non-affective psychotic disorders (mean age 32 years) with a maximum duration of illness of ten years and 32 control subjects without a diagnosis of psychotic illness [15]. An evaluation of mean-level fluctuations among patients and controls revealed no significant differences between baseline and follow-up, with the exception of Conscientiousness among patients. Using Spearman correlations the relationships between baseline and follow-up were moderate to strong (rho 0.51–0.68) and moderate to very strong (rho 0.57–0.90) among patients and controls, respectively. Results showed that psychotic symptoms have a limited effect on the stability of FFM traits in patients with psychotic disorders.

Horan and colleges analysed intra-class correlations (ICCs) of the MMPI personality traits Neuroticism, Denial of somatic complaints, Cynicism, Psychopathic tendencies and Psychotic ideation among patients with schizophrenia spectrum disorders (mean age 23 years) and control subjects investigated three times during a period of 15 months [16]. These authors found ICCs between 0.29 to 0.55 for patients and 0.66 to 0.83 for controls, indicating less stability among patients than controls. Nonetheless, considerable stability of personality traits was indicated among patients.

Swedish universities scales of personality (SSP) is an established personality instrument, preferably used in Sweden and other countries in northern Europe since 17 years. It is an elaboration of the instrument Karolinska Scales of Personality (KSP), which was designed to measure stable personality traits related to psychopathology [17, 18]. SSP has been described in detail previously [19]. In contrast to other personality instruments translated into Swedish, such as the NEO-PI-R or the Temperament and Character Inventory (TCI) [20], SSP can be used for free in the clinic. SSP gives comparison possibilities versus the general population for 13 different clinically relevant personality aspects. This together with its relatively short format (91 items, compared to 238–240 items for the full NEO and TCI questionnaires) has made it common in clinical practice in Sweden. SSP has been subjected to factor analysis and comprise the following higher order factors: Neuroticism, Aggressiveness and Extraversion [19, 21]. When compared with the FFM using NEO-PI-R Aluoja and coworkers (2009) described as an overall pattern, although with some exceptions, that SSP neuroticism-related scales had their highest loadings on NEO Neuroticism, SSP extraversion-related scales on NEO Extraversion and the Aggression-related scales on NEO Agreeableness [21]. However, SSP has until recently not been used among psychotic patients within research [22]. Also SSPs predecessor KSP has only sparsely been evaluated among patients with psychosis [23, 24], although both SSP and KSP have been frequently used among other psychiatric patient categories.

Three previous studies have investigated stability of personality traits measured using the SSP at two different time-points. A Swedish research team investigated the impact of internet-based cognitive behaviour therapy (ICBT) on personality traits [25]. In a randomized controlled trial participants with a mean age of 39 years were distributed to 12 weeks of ICBT (*n* = 40) or to a basic attention control condition (*n* = 41). During the latter condition patients scored essentially similar at baseline and at follow-up (z-score deviation < 0.21 in all 13 SSP-scales). However, ICBT patients scored lower on neuroticism-related scales with z-scores deviating up to 0.48 (Psychic trait anxiety) and 0.52 (Somatic trait anxiety) in the second compared to the first assessment, whereas for the non-neuroticism-related scales no difference exceeded z = 0.25.

Estonian researchers investigated 107 patients (mean age 34 years) with panic disorder before and after 12 weeks of treatment with escitalopram [26]. There were no significant differences between SSP baseline and post-treatment scale scores, although almost all scores being abnormal from the beginning were less deviant after treatment. The largest changes occurred for the neuroticism-related scales Somatic trait anxiety (z = 0.67), Psychic trait anxiety (z = 0.53) and Stress susceptibility (z = 0.48), whereas for the remaining SSP scales the differences were below z = 0.35.

In an eight weeks follow-up study possible effects of mechanical massage, mental training programmes or a combination of interventions on healthy employees with a mean age of 48 years were examined by using the SSP personality traits Somatic Trait Anxiety (STA), Psychic Trait Anxiety (PsTA), Stress Susceptibility (SS), Detachment (D) and Social Desirability (SD) [27]. Employees were randomly distributed to one of five intervention groups; Massage and mental training, Massage, Mental training, Pause and Control with 13 to 18 participants in each group at the end of the trial. SSP mean z-score deviations between baseline and eight weeks of intervention were for STA − 0.19, PsTA -0.09, SS -0.09, D 0.17 and SD 0.17, and with no z-score deviation exceeding 0.4 in the various small intervention groups.

Stability in personality traits in patients with psychotic disorder has not previously been investigated at time intervals longer than three years and with participants with a mean age above 34 years. In the present study we investigated outpatients diagnosed with a psychotic disorder and non-psychotic control subjects on two occasions five years apart, with a mean age at the first investigation of about 40 years. We used SSP [19], a questionnaire not previously assessed for stability measures of personality for this time frame and in this patient category.

We aimed to investigate firstly, whether personality traits were stable over a five-year period in a sample of patients with psychosis; and secondly whether patients with psychosis differed from non-psychotic individuals.

## Methods

### Participants

Patients diagnosed with long term psychotic disorder were recruited from outpatient clinics treating individuals with psychotic disorders. These clinics were located in the North-Western part of Stockholm County. The patients were diagnosed according to DSM-III-R and DSM-IV based on information from interviews and medical records as previously described [28, 29]. Non-psychotic siblings of patients with psychosis were asked to participate when their relative with a psychotic disorder had agreed to their participation. Control subjects were recruited among students, hospital staff members or from a population register. All controls with the exception of those recruited from a population register had earlier attended in biological research at the Karolinska Institute [22, 30–32]. The controls consisted of non-psychotic individuals unrelated to the patients. Neither the siblings, nor the controls received psychotic diagnosis according to DSM-III-R and DSM-IV. Patients and controls were interviewed by a psychiatrist who administered the Structured Clinical Interview for DSM-III-R, axis I (SCID-I) [33], the psychosis module (chapters 17–19) of the Schedules for Clinical Assessment in Neuropsychiatry [34], the Scales for assessment of negative symptoms (SANS) [35], the Scales for assessment of positive symptoms (SAPS) [36], the Global assessment of functioning (GAF) scale [37], as well as questions about demography, medication, somatic illness, and family history of mental illness. In the end of the interview with the psychiatrist subjects were asked to fill in the personality questionnaire while still at the psychiatrist’s office. The psychiatrist checked the completed questionnaire and asked the participants to fill in any items that have not been answered. In addition participants were subjected to neuropsychological testing [38], performed by a psychologist or a psychology resident, magnetic resonance imaging of the brain [39], and blood sampling for genetic investigations and routine analyses. These additional investigations were often, but not always performed on the same day as the clinical interview. Usually all investigations were performed at the Karolinska Hospital, although occasionally the psychiatrist performed the interview in the outpatient clinic or the home of the patient. Only subjects who participated both at a baseline investigation performed between 1999 and 2003 and a follow-up investigation about five years later were included. At baseline 107 patients and 142 non-psychotic individuals were investigated, of whom 36 and 76, respectively, participated at follow-up.

### Psychometric instruments

The self-report questionnaire SSP consists of 91 items grouped into 13 different scales [19] (with item examples within brackets); Somatic Trait Anxiety (STA; My body often feel stiff and tense), Psychic Trait Anxiety (PsTA; I am the kind of person who is excessively sensitive and easily hurt), Stress Susceptibility (SS; I get tired and hurried to easily), Lack of Assertiveness (LA; Even though I know I am right I often have great difficulty getting my points across), Detachment (D; I feel best when I keep people at a certain distance), Embitterment (E; I have often got into trouble even when it was not my fault), Mistrust (M; I tend to be on my guard with people who are somewhat more friendly than I expected), Physical Trait Aggression (PhTA; If someone hits me, I hit back), Verbal Trait Aggression (VTA; When I get angry, I often express myself ironically or sarcastically), Adventure Seeking (AS; I have an unusually great need for change), Impulsiveness (I; I have a tendency to act on the spur of the moment without really thinking ahead), Social Desirability (SD; No matter whom I am talking to, I am always polite and courteous), and Trait Irritability (TI; I do not have so much patience). Participants were asked to endorse one of four alternatives in relation to each of the 91 items. Reliability and validity of SSP, in terms of repeated measures within the same individual with similar results [25–27], similar internal consistencies [19, 21, 22, 25], similar factor loadings between different samples [19, 21, 22, 40, 41] and conformity with other personality constructs has been documented [21].

Function was measured by the GAF scale [37]. To assess the verbal intelligent quotient (IQ) the vocabulary part of Wechsler adult intelligence scales (WAIS) [42] was used. SANS and SAPS [35, 36] were used to assess psychotic symptoms. Chlorpromazine equivalents [43] were used for an overall estimate of consumption of antipsychotics.

### Data analyses

The SSP manual was used to calculate the 13 personality scales from the 91 items common to both the SSP and KSP-196 questionnaires.

Internal consistency was evaluated using Cronbach’s alpha [44]. Statistical power was evaluated for a paired samples *t*-test for patients (*n* = 36) and non-psychotic individuals (*n* = 76) separately, with the mean difference expressed as a non-trivial z-value (z_critical_ = 0.5) [45, 46] and α = 0.05, given that an approximate estimate suffices [47]. The statistical power was computed using G*Power (version 3) freeware power calculator [48].

Subjects were initially divided into three groups: patients (*n* = 36), their non-psychotic siblings (*n* = 17) and non-psychotic controls (*n* = 59). In order to control for multiple testing the statistical analysis of the 13 SSP-scales was performed using multiple analysis of covariance (MANCOVA) with diagnosis (psychosis vs siblings vs controls) and gender (male vs female) as between-subject factors, time (baseline vs follow-up) as within-subject factor, and age as a covariate. This preliminary analysis did not show any significant differences between siblings and controls (data not shown). Therefore, in order to simplify the presentation siblings and controls were pooled into one group of non-psychotic individuals. MANCOVA was redone with diagnosis (psychosis vs non-psychosis) and gender (male vs female) as between-subject factors, time (baseline vs follow-up) as a within-subject factor and age as a covariate. As a third step the corresponding ANCOVAs were performed for each SSP-scale separately.

To further illustrate the effect of time linear and rank-order correlations according to Pearson (r) and Spearman (rho), respectively, as well as intraclass correlations (ICC) were calculated between baseline and follow-up scores for each of the 13 SSP scales for patients with psychosis and non-psychotic individuals. ICC coefficients for single measures were calculated both for consistency and agreement. In order to analyse the test-retest correlations in the same manner as the mean of the 13 SSP-scales, the correlations were jackknifed [49]. This was done by recomputing the correlations excluding one individual at a time, resulting in (36 + 76)*13 estimates. Before performing the final J-summaries, all correlations (Pearson) were transformed to an approximate normal distribution, using the Z-transform, producing a better statistical estimate. The computations were performed using a special purpose program. Formulas are given below:

$$ \mathrm{J}={\mathrm{n}}^{\ast }{\mathrm{Z}}_{\mathrm{total}}\hbox{--} \left(\mathrm{n}-1\right)\ast {\mathrm{Z}}_{\left(-1\right)} $$

$$ \mathrm{Z}={0.5}^{\ast}\mathrm{elog}\left(\left(1+\mathrm{r}\right)/\left(1-\mathrm{r}\right)\right) $$

The z-transformed and normalized correlations were analyzed in SPSS in a similar manner as the analyses of the means: first MANCOVA with diagnosis (psychosis vs non-psychosis) and gender (male vs female) as between-subject factors and age as a covariate followed by ANCOVAs for each of the SSP-scales. SPSS version 17.0.1 for Windows, IBM software was used for statistical analyses.

## Results

### Characterisation of subjects

Characteristics of the subjects are shown in Table 1. There were eight (22%) female and 28 (78%) male patients with psychotic disorder and 29 (38%) women and 47 (62%) men among the non-psychotic subjects (chi squared = 2.130, *p* = 0.144). The mean age (SD; range) was at baseline among female patients 37.5 (8.2; 25–50), male patients 36.9 (7.5; 24–50), female control subjects 40.8 (7.4; 24–50), and male control subjects 41.2 (8.1; 23–53) years, respectively. No significant case-control differences with regard to age or gender was found (Table 1). Patients had a lower level of functioning, lower verbal IQ and tended to be less educated (Table 1). Mean age at onset of illness was 24.2 years. Patients were diagnosed with psychosis not otherwise specified (*n* = 3), schizoaffective disorder (*n* = 7) and schizophrenia (*n* = 26).

**Table 1:** Characteristics of patients and controls

	Patients (*n* = 36)		Controls (*n* = 76)	
	Baseline	Follow-up	Baseline	Follow-up
Gender (n, women/men)	8/28	8/28	29/47	29/47
Age (year)	39.4	44.4	41.1	47.5§
Education (year)	12.6	NA	14.0§	NA
WAIS verbal IQ	87.3	NA	103.0***	NA
GAF	49.2	48.3	87.3***	83.9***
SANS composite score	28.9	33.4	NA	NA
SAPS composite score	10.0	11.1	NA	NA
Medication (mg, CPZ-equivalents)	208.9	263.3	NA	NA
Medication-no antipsychotics (n)	4	5	NA	NA
Medication-2nd gen antipsychotics (n)	14	15	NA	NA
Medication-1st gen antipsychotics (n)	16	9	NA	NA
Medication-1st and 2nd gen antipsychotics (n)	2	7	NA	NA

*NS* not significant, *NA* not applicable or not assessed, *WAIS* Wechsler adult Intelligence Scales, *IQ* intelligent quotient, *GAF* Global Assessment of Functioning, *SANS* Scale for the Assessment of Negative Symptoms, *SAPS* Scale for the Assessment of Positive Symptoms, *CPZ* chlorpromazine, *gen* generation, *NOS* not otherwise specified. All values in mean (standard deviation) except for distribution of gender, diagnosis and medication

Missing data (patients/controls): Education (0/1), WAIS vocabulary (7/12), GAF baseline (0/2), GAF follow-up (0/7), SANS/SAPS baseline (8/NA)

§*p* < 0.1 ***p < 0.001

### Internal consistency

The attrition rate was among male cases 0.72, female cases 0.77, non-psychotic men 0.53 and non-psychotic women 0.54. Among the 71 patients who did not participate at follow-up the reasons were: declined to participate or no contact (*n* = 54), dead (*n* = 15), changed residence to a region far away or emigrated (*n* = 2). Reasons for drop-out among the 66 controls were: no available time (*n* = 5), declined to participate or no contact (*n* = 54), changed residence to a region far away or emigrated (*n* = 7). Subjects participating and not participating at follow-up did not significantly differ at baseline with regard to gender, age, verbal IQ, GAF, negative or positive psychotic symptomatology, chlorpromazine equivalent dose of antipsychotic medication or any of the SSP personality traits.

Internal consistency of the 13 SSP-scales was evaluated at both baseline and follow-up for patients and non-psychotic individuals separately (Table 2). Consistencies were above 0.70 for 54% of the patients and 82% of the non-psychotic individuals. Consistencies were above 0.60 for 81% of the patients and 96% of the non-psychotic individuals. Among patients Somatic Trait Anxiety, Stress Susceptibility, Impulsiveness, Detachment, Social Desirability, Embitterment and Mistrust and among non-psychotic individuals Lack of Assertiveness and Social Desirability was below 0.70 for at least one of the time-points.

**Table 2:** Swedish Scales of Personality (SSP) internal consistency data given as Cronbach’s α for psychotic patients (*n* = 36) and control subjects (*n* = 76) at baseline and follow-up

SSP scale	Patients		Controls	
	Baseline	Follow-up	Baseline	Follow-up
Somatic Trait Anxiety	0.45	0.52	0.72	0.74
Psychic Trait Anxiety	0.75	0.73	0.85	0.77
Stress Susceptibility	0.69	0.63	0.80	0.78
Lack of Assertiveness	0.77	0.77	0.65	0.71
Impulsiveness	0.64	0.59	0.74	0.76
Adventure Seeking	0.72	0.74	0.82	0.84
Detachment	0.79	0.60	0.77	0.75
Social Desirability	0.66	0.54	0.64	0.56
Embitterment	0.56	0.66	0.78	0.77
Trait Irritability	0.76	0.80	0.79	0.77
Mistrust	0.78	0.68	0.86	0.84
Verbal Trait Aggression	0.80	0.72	0.73	0.74
Physical Trait Aggression	0.80	0.80	0.87	0.84

### Effect by time on mean differences

For an overview of the personality data at baseline and follow-up, see Table 3. Controls showed nominally higher estimates of Social Desirability and lower estimates of Psychic Trait Anxiety, Impulsiveness, Adventure Seeking, Embitterment, Trait Irritability, Mistrust, Verbal Trait Aggression and Physical Trait Aggression at follow-up compared to baseline. Among patients Adventure Seeking and Trait Irritability showed a similar pattern as in the non-psychotic individuals. However, after correction for multiple testing and taking covariates into account, MANCOVA within-subjects analysis of the means did not show any significant effect of time, interaction time * age, interaction time * diagnosis, interaction time * gender, or interaction time * diagnosis * gender (all Wilk’s lambda> 0.825, all *p* > 0.117).

**Table 3:** Swedish universities Scales of Personality (SSP) raw scores (mean [standard deviation]) and 95% confidence interval (CI) of the difference between baseline and follow-up in patients with psychotic illness and non-psychotic controls

			Somatic Trait Anxiety			Psychic Trait Anxiety			Stress Susceptibility		
		N	Baseline	Follow-up	CI	Baseline	Follow-up	CI	Baseline	Follow-up	CI
ALL	Patient	36	39.4 (7.7)	44.4 (7.8)	−0.25 – 0.15	2.08 (0.48)	2.13 (0.53)	−0.09 – 0.27	2.45 (0.62)	2.36 (0.61)	−0.19 – 0.16
	Control	76	41.1 (7.8)	47.5 (7.8)	−0.06 – 0.12	1.58 (0.46)	1.55 (0.45)	0.11–0.26	1.66 (0.50)	1.48 (0.39)	−0.05 – 0.14
ALL	Men	75	40.7 (7.9)	46.5 (8.0)	−0.14 – 0.08	1.68 (0.48)	1.71 (0.56)	0.04–0.22	1.92 (0.68)	1.79 (0.66)	−0.06 – 0.14
	Women	37	40.1 (7.7)	46.4 (7.9)	−0.08 – 0.21	1.74 (0.52)	1.78 (0.53)	0.07–0.34	1.90 (0.60)	1.70 (0.55)	−0.17 – 0.16
PATIENT	Men	28	39.9 (7.5)	44.9 (7.6)	−0.39 – 0.09	2.05 (0.39)	2.19 (0.51)	−0.17 – 0.24	2.47 (0.60)	2.43 (0.52)	−0.24 – 0.17
	Women	8	37.5 (8.2)	42.6 (8.2)	0.02–0.55	2.21 (0.71)	1.93 (0.57)	−0.17 – 0.74	2.38 (0.71)	2.09 (0.84)	−0.40 – 0.51
CONTROL	Men	47	41.2 (8.1)	47.5 (8.0)	−0.06 – 0.14	1.47 (0.39)	1.43 (0.36)	0.10–0.28	1.60 (0.49)	1.41 (0.37)	−0.02 – 0.20
	Women	29	40.8 (7.4)	47.5 (7.5)	−0.16 – 0.18	1.75 (0.71)	1.74 (0.52)	0.04–0.33	1.77 (0.51)	1.59 (0.40)	−0.21 – 0.17
		Lack of Assertiveness			Impulsiveness			Adventure Seeking			
		Baseline	Follow-up	CI	Baseline	Follow-up	CI	Baseline	Follow-up	CI	
ALL	Patient	2.46 (0.55)	2.47 (0.53)	−0.13 – 0.28	2.33 (0.52)	2.25 (0.51)	−0.07 – 0.23	2.50 (0.58)	2.38 (0.61)	10.04–0.29	
	Control	1.87 (0.47)	1.82 (0.44)	−0.04 – 0.11	2.30 (0.46)	2.19 (0.47)	0.02–0.20	2.53 (0.52)	2.39 (0.51)	0.07–0.22	
ALL	Men	2.06 (0.61)	2.02 (0.58)	−0.06 – 0.16	2.23 (0.47)	2.15 (0.47)	−0.01 – 0.17	2.57 (0.58)	2.42 (0.56)	0.06–0.25	
	Women	2.04 (0.46)	2.05 (0.53)	−0.07 – 0.15	2.48 (0.45)	2.34 (0.49)	0.01–0.28	2.42 (0.43)	2.33 (0.50)	−0.01 – 0.21	
PATIENT	Men	2.48 (0.60)	2.52 (0.46)	−0.18 – 0.33	2.28 (0.51)	2.21 (0.52)	−0.12 – 0.24	2.52 (0.62)	2.34 (0.65)	−0.02 – 0.38	
	Women	2.36 (0.38)	2.30 (0.76)	−0.21 – 0.35	2.52 (0.51)	2.38 (0.48)	−0.22 – 0.49	2.46 (0.39)	2.54 (0.46)	−0.33 – 0.19	
CONTROL	Men	1.81 (0.47)	1.72 (0.41)	−0.06 – 0.13	2.19 (0.44)	2.11 (0.44)	−0.02 – 0.20	2.61 (0.55)	2.47 (0.51)	0.04–0.24	
	Women	1.96 (0.45)	1.98 (0.44)	−0.09 – 0.16	2.47 (0.44)	2.33 (0.50)	−0.00 – 0.30	2.41 (0.44)	2.27 (0.50)	0.02–0.27	
		Detachment			Social Desirability			Embitterment			
		Baseline	Follow-up	CI	Baseline	Follow-up	CI	Baseline	Follow-up	CI	
ALL	Patient	2.77 (0.53)	2.83 (0.45)	−0.14 – 0.17	2.77 (0.53)	2.83 (0.45)	−0.24 – 0.13	2.23 (0.50)	2.22 (0.55)	−0.15 – 0.17	
	Control	2.83 (0.37)	2.92 (0.35)	−0.02 – 0.12	2.83 (0.37)	2.92 (0.35)	−0.15 - -0.03	1.60 (0.41)	1.47 (0.40)	0.05–0.20	
ALL	Men	2.80 (0.43)	2.88 (0.39)	−0.05 – 0.12	2.80 (0.43)	2.88 (0.39)	−0.16 – 0.00	1.82 (0.52)	1.72 (0.59)	0.01–0.20	
	Women	2.82 (0.42)	2.90 (0.38)	−0.09 – 0.18	2.82 (0.42)	2.90 (0.38)	−0.21 – 0.06	1.75 (0.56)	1.69 (0.59)	−0.06 – 0.18	
PATIENT	Men	2.82 (0.56)	2.80 (0.46)	−0.14 – 0.20	2.82 (0.56)	2.80 (0.46)	−0.18 – 0.21	2.23 (0.44)	2.23 (0.57)	−0.20 – 0.20	
	Women	2.61 (0.42)	2.91 (0.44)	−0.51 – 0.48	2.61 (0.42)	2.91 (0.44)	−0.87 – 0.26	2.23 (0.69)	2.17 (0.51)	−0.22 – 0.34	
CONTROL	Men	2.79 (0.34)	2.93 (0.34)	−0.05 – 0.13	2.79 (0.34)	2.93 (0.34)	−0.21 - − 0.07	1.58 (0.39)	1.42 (0.34)	0.08–0.25	
	Women	2.88 (0.41)	2.90 (0.36)	-0.07 – 0.20	2.88 (0.41)	2.90 (0.36)	−0.12 – 0.09	1.62 (0.45)	1.56 (0.47)	−0.08 – 0.20	
		Trait Irritability				Mistrust					
		Baseline	Follow-up	CI		Baseline	Follow-up	CI			
ALL	Patient	2.35 (0.57)	2.13 (0.57)	0.03–0.40		2.26 (0.58)	2.26 (0.52)	−0.13 – 0.15			
	Control	2.21 (0.48)	2.03 (0.49)	0.09–0.26		1.75 (0.50)	1.60 (0.47)	0.06–0.24			
ALL	Men	2.26 (0.49)	2.04 (0.53)	0.12–0.31		1.97 (0.56)	1.86 (0.57)	0.02–0.21			
	Women	2.24 (0.55)	2.11 (0.50)	−0.03 – 0.29		1.81 (0.59)	1.72 (0.58)	−0.03 – 0.21			
PATIENT	Men	2.36 (0.56)	2.16 (0.53)	0.01–0.38		2.28 (0.58)	2.25 (0.53)	−0.14 – 0.21			
	Women	2.32 (0.66)	2.04 (0.74)	−0.37 – 0.94		2.20 (0.62)	2.29 (0.55)	−0.24 – 0.07			
CONTROL	Men	2.20 (0.45)	1.97 (0.51)	0.11–0.34		1.79 (0.47)	1.62 (0.47)	0.05–0.28			
	Women	2.22 (0.48)	2.13 (0.43)	−0.05 – 0.22		1.70 (0.54)	1.56 (0.49)	−0.01 -0.29			
		Verbal Trait Aggression				Physical Trait Aggression					
		Baseline	Follow-up	CI		Baseline	Follow-up	CI			
ALL	Patient	2.01 (0.64)	2.00 (0.55)	−0.19 – 0.20		1.71 (0.57)	1.84 (0.60)	−0.31 – 0.07			
	Control	2.15 (0.47)	1.96 (0.46)	0.10–0.28		1.93 (0.57)	1.67 (0.54)	0.17–0.35			
ALL	Men	2.12 (0.53)	2.02 (0.58)	−0.01 – 0.21		1.92 (0.60)	1.81 (0.59)	0.00–0.23			
	Women	2.07 (0.55)	1.88 (0.43)	0.04–0.33		1.73 (0.53)	1.55 (0.46)	0.01–0.33			
PATIENT	Men	2.03 (0.62)	2.07 (0.58)	−0.26 – 0.18		1.76 (0.59)	1.86 (0.66)	−0.33 – 0.13			
	Women	1.95 (0.74)	1.79 (0.38)	−0.37 – 0.69		1.54 (0.47)	1.74 (0.39)	−0.57 – 0.16			
CONTROL	Men	2.18 (0.46)	2.00 (0.48)	0.07–0.31		2.02 (0.58)	1.78 (0.55)	0.14–0.35			
	Women	2.10 (0.50)	1.91 (0.44)	0.04–0.34		1.78 (0.54)	1.50 (0.47)	0.11–0.44			

Data is shown by diagnosis (patient vs control) in all subjects, gender in all subjects, gender among patients and gender among controls

### Effect by time on interpersonal correlations

Within-subjects correlations are shown in Table 4. For patients the rank-order correlations (rho) between the two testing time-points varied between 0.30 and 0.68, with the lowest correlations for Somatic Trait Anxiety (0.30) and Social Desirability (0.38) and the highest for Mistrust (0.68). Among non-psychotic individuals the correlations varied between 0.54 (Stress Susceptibility) and 0.75 (Adventure Seeking). Linear correlations (r) and ICCs were of a similar magnitude (Table 4). The MANCOVA analysis of the test-retest correlations was significant with regard to diagnosis (Wilk’s lambda 0.791, *p* = 0.036) but not to gender (Wilk’s lambda 0.825, *p* = 0.114), age (Wilk’s lambda 0.848, *p* = 0.222) or interaction between diagnosis and gender (Wilk’s lambda 0.826, *p* = 0.119). In post-hoc analyses ANCOVAs were performed for each of the SSP scales. In the 13 post-hoc ANCOVAs of the test-retest correlations the following nominal differences was found: age and Detachment (*F* = 4.2, *p* = 0.044), age and Social Desirability (*F* = 4,9, *p* = 0.029), diagnosis and Detachment (*F* = 11.3, *p* = 0.001), gender and Social Desirability (*F* = 4.5, *p* = 0.035), gender * diagnosis and Detachment (*F* = 4.3, *p* = 0.040), and gender * diagnosis and Social Desirability (*F* = 5.8, *p* < 0.018).

**Table 4:** Correlations between baseline and five year follow-up assessments of the Swedish universities Scales of Personality (SSP) in psychotic patients (*n* = 36) and control subjects (*n* = 76)

SSP scale	Spearman (rho)		Pearson (r)		ICC Consistency		ICC Agreement	
	Patients	Controls	Patients	Controls	Patients	Controls	Patients	Controls
Somatic Trait Anxiety	0.30	0.57	0.31	0.64	0.30	0.64	0.31	0.64
Psychic Trait Anxiety	0.61	0.73	0.63	0.75	0.62	0.73	0.62	0.67
Stress Susceptibility	0.59	0.54	0.53	0.57	0.53	0.57	0.54	0.57
Lack of Assertiveness	0.48	0.64	0.56	0.70	0.56	0.70	0.56	0.70
Impulsiveness	0.62	0.64	0.62	0.67	0.62	0.67	0.62	0.65
Adventure Seeking	0.61	0.75	0.67	0.79	0.67	0.79	0.66	0.76
Detachment	0.67	0.63	0.69	0.71	0.67	0.71	0.68	0.71
Social Desirability	0.38	0.70	0.38	0.75	0.38	0.75	0.38	0.73
Embitterment	0.56	0.70	0.59	0.68	0.58	0.68	0.59	0.65
Trait Irritability	0.51	0.70	0.55	0.70	0.55	0.70	0.52	0.66
Mistrust	0.68	0.66	0.72	0.67	0.72	0.67	0.78	0.64
Verbal Trait Aggression	0.46	0.58	0.53	0.64	0.52	0.64	0.53	0.59
Physical Trait Aggression	0.52	0.69	0.54	0.75	0.54	0.75	0.54	0.68

ICC koefficients are single measures both for Consistency and Agreement

### Between-subject analyses

For the analysis of mean differences between psychotic patients and non-psychotic individuals MANCOVA between-subjects analysis was significant with regard to diagnosis (Wilk’s lambda 0.563, *p* < 0.001) and gender (Wilk’s lambda 0.682, *p* < 0.001) but not to age (Wilk’s lambda 0.908, *p* = 0.715) or interaction between diagnosis and gender (Wilk’s lambda 0.873, *p* = 0.402). In post-hoc analyses ANCOVAs were performed for each of the SSP scales. For seven of the scales, i.e. Somatic trait anxiety (*F* = 26.0, *p* < 0.001), Psychic trait anxiety (*F* = 43.9, *p* < 0.001), Stress Susceptibility (*F* = 28.7, *p* < 0.001), Lack of Assertiveness (*F* = 20.6, *p* < 0.001), Detachment (*F* = 14.6, *p* < 0.001), Embitterment (*F* = 45.5, *p* < 0.001), and Mistrust (*F* = 25.0, *p* < 0.001) patients scored significantly higher than controls, whereas for six (Impulsiveness [*F* = 0.8, *p* = 0.371], Adventure seeking [*F* = 0.2, *p* = 0.650]; Social desirability [*F* = 0.9, *p* = 0.345], Trait Irritability [*F* = 0.2, *p* = 0.621], Verbal Trait Aggression [*F* = 1.3, *p* = 0.27], Physical Trait Aggression [*F* = 0.3, *p* = 0.609]) no significant differences were found. Age effects were found for Psychic Trait Anxiety (*F* = 3.8, *p* = 0.049), gender effects were found for Impulsiveness (*F* = 4.8, *p* = 0.031) and Detachment (*F* = 11.7, *p* = 0.001) and interaction diagnosis * gender effect was found for Somatic Trait Anxiety (*F* = 3.9, *p* = 0.050).

### Power

The statistical power was computed using the G*Power (version 3) freeware Power calculator [48]. Given α = 0.05 and a mean difference of z = 0.5, the sample of patients (*n* = 36) had a power of 83% whereas the sample of non-psychotic individuals (*n* = 76) had a power of 99%.

## Discussion

The main finding of the present study is that SSP mean scale scores did not vary significantly during the five-year interval. Within-subjects correlations showed less stability for the rank order between individuals for some of the scales, especially among patients. The stability of the SSP personality traits was reasonably high among patients with psychotic disorder, although lower than among non-psychotic individuals. This is in accordance with previous studies using the Five-Factor Model and MMPI [13–16].

We know about three previous studies in which the same individuals have been investigated with the SSP inventory at two different time-points [25–27]. With regard to stability over time these three studies give overall similar results as the present study, despite different time span (two – three months versus five years), different ages and different patient categories investigated. The z-score deviation between baseline and follow-up in the present study never exceeded 0.5, which is considered as a lower limit for a non-trivial difference [45, 46]. With the exception of Somatic Trait Anxiety in the ICBT-intervention group in the study of health anxiety [25] and Somatic Trait Anxiety and Psychic Trait Anxiety among the escitalopram-treated Estonian patients with panic disorder [26] this was not the case in the other studies. The most deviant z-scores in the present study was among controls who scored lower on Psychic Trait Anxiety (− 0.40), Verbal Trait Aggression (− 0.41) and Physical Trait Aggression (− 0.47) at the five-year follow-up than at baseline**,** whereas among patients the most deviant measures was that of Trait Irritability (− 0.39). This indicates that the mean SSP trait scores evaluated at different time-points do not differ substantially and that SSP scale scores in general are stable over time periods up to five years.

In the present study there were differences between psychotic patients and non-psychotic individuals with regard to the stability of interpersonal correlations for two SSP scales: Detachment and Social Desirability. In an attempt to explain these differences we analysed the impact of verbal IQ (WAIS verbal IQ) and functioning (GAF) by correlation with the five-year interpersonal correlations of Detachment and Social Desirability. We also analyzed if psychotic negative (SANS) or positive symptoms (SAPS) were associated with Detachment and Social Desirability interpersonal correlations among patients. However, we did not find any significant association for either of these analyses (data not shown). Thus, we were not able to find the reason why the stability differed between patients and controls for these two SSP scales. The sample was small, and therefore we may have had too little power to detect a difference. It is also possible that these differences may have occurred by chance or that the differences were due to other factors not accounted for in our study. Interestingly, Kentros and co-workers [13] found a lower stability with regard to NEO Agreeableness in patients with psychosis. SSP Social Desirability has been shown to be associated with NEO Agreeableness [21], indicating some concordance between the study of Kentros and co-workers and the present study.

Kentros and collaborators [13] reported overall stability of personality traits, despite substantial variation with regard to psychotic symptoms. Beauchamp et al. [14] found both results in favour of and in disagreement with the idea that personality is associated with psychotic symptoms over a three-month period, whereas Boyette and co-workers [15] reported lower three-year correlations among psychotic patients than controls with regard to NEO Neuroticism, which was associated with depressive symptoms. Thus, stability with regard to the Five-Factor Model among psychotic patients seems to be overall steady, with a few exceptions, similar to the results of the present study using SSP. In contrast, among the MMPI personality traits analysed by Horan and collaborators over a 15-month period the majority was influenced by psychiatric symptoms [16]. In this context, the attempts to overcome shortcomings in traditional psychopathological taxonomy by incorporating personality variation, partly overlapping with aspects of psychopathology is of interest [50].

### Case-control differences

In this study patients scored higher than controls in six neuroticism-related SSP-scales, i.e. Somatic Trait Anxiety, Psychic Trait Anxiety, Stress Susceptibility, Lack of Assertiveness, Embitterment and Mistrust, as well as Detachment, a negative facet of extraversion. This is consistent with a previous study [22], from which the majority of the present research individuals constitute a sub-sample. Given this fact and the relative stability of the personality traits over time this is anticipated. The results are also in agreement with the majority of previous studies using other personality instruments that indicate that facets of neuroticism are particularly prominent in patients with psychotic disorders [3, 8, 10, 11, 23, 24].

### Strength and limitations

One limitation of the current study is that the participants had agreed to take part in an extensive biological research. This makes neither the group with patients nor the healthy subjects fully representative. The presence of a non-psychotic control group, involved in the same demanding research as the patients, is on the other hand a strength of the study. Another limitation is the high attrition rate, making the number of participants included in the study limited, which may have reduced our possibility to find age-related personality changes. High drop-out rates is unfortunately common in follow-up studies performed several years apart. This may have given our sample more subjects with non-significant tendencies to getting worse over time, making the conclusions less valid for subjects without such tendencies. Another limitation was the relatively small number of women, giving rise to a gender disparity in the group of patients relative to the overall reported gender distribution of psychotic disorders in the population [51]. Participants in the present study were adults in the middle age (mean age at baseline about 40 years), a part of life known to be relatively stable with regard to most personality traits in the general population [12]. Also the previous studies [13–16] analyzing personality over time in patients with psychosis studied adult patients but at lower ages (mean ages at baseline from 23 to 34 years), although after the adolescence period. The age ranges may thus have diminished the possibility to detect differences in all these studies. The patients were not investigated with any instrument assessing personality disorder. Non-psychotic individuals were however given the SCID-II screen questionnaire [52] at baseline but there was none fulfilling suggested criteria for any personality disorder. The lack of assessment of personality disorder diagnoses adds to the limitations of the study. The SSP, used in the present study, is not identical with the personality inventories used in previous studies. This complicates the ability to compare the current study with previous studies. However, SSP captures several of the personality aspects that are measured by other questionnaires and may have special advantages as the relatively small numbers of items comparing to other personality constructs and the use of a simple and timeless language. Some scales in SSP and its predecessors also appear to have biological relevance [53–55].

## Conclusions

SSP mean scale scores did not significantly vary during a five-year interval. The stability of the SSP personality traits was reasonably high among patients with psychotic disorders, although lower than among non-psychotic individuals. This is in accordance with previous studies using other personality instruments. Data must be interpreted with caution because of a high number of participants lost to follow-up.

## Abbreviations

ANCOVA: analysis of covariance
AS: Adventure Seeking
D: Detachment
DSM-III-R: Diagnostic and Statistical Manual of Mental Disorders, 3th version, Revised
DSM-IV: Diagnostic and Statistical Manual of Mental Disorders, 4th version
E: Embitterment
FFM: Five-Factor Model
GAF: Global assessment of functioning
I: Impulsiveness
ICBT: internet-based cognitive behaviour
ICC: intraclass correlations
IQ: intelligent quotient
KSP: Karolinska Scales of Personality
LA: Lack of Assertiveness
M: Misstrust
MANCOVA: multiple analysis of covariance
MMPI-168: Minnesota Multiphasic Personality Inventory
NEO-FFI: NEO-Five Factor Inventory
NEO-PI: NEO-Personality Inventory
NEO-PI-R: NEO-Personality Inventory Revised
PhTA: Physical Trait Aggression
PsTA: Psychic Trait Anxiety
SANS: Scales for assessment of negative symptoms
SAPS: Scales for assessment of positive symptoms
SD: Social Desirability
SS: Stress Susceptibility
SSP: Swedish universities Scales of Personality
STA: Somatic Trait Anxiety
TI: Trait Irritability
VTA: Verbal Trait Aggression
WAIS: Wechsler adult intelligence scales
