# Correction to: Stability of personality traits over a five-year period in Swedish patients with schizophrenia spectrum disorder and nonpsychotic individuals: a study using the Swedish universities scales of personality

**DOI:** 10.1186/s12888-019-2086-7

**Published:** 2019-04-08

**Authors:** Tomas Fagerberg, Erik Söderman, J. Petter Gustavsson, Ingrid Agartz, Erik G. Jönsson

**Affiliations:** 1 Human Brain Informatics (HUBIN), Department of Clinical Neuroscience, Centre for Psychiatric Research, Psychiatry Section, Karolinska Institutet and Hospital, Stockholm, Sweden; 2 Division of Psychology, Department of Clinical Neuroscience, Karolinska Institutet, Stockholm, Sweden; 3 NORMENT, KG Jebsen Centre for Psychosis Research, Institute of Clinical Medicine, Psychiatry Section, University of Oslo, Oslo, Norway; 4 Department of Psychiatric Research, Diakonhjemmet Hospital, Oslo, Norway

**Correction to: BMC Psychiatry**

**https://doi.org/10.1186/s12888-018-1617-y**

After publication of the original article [1], it was brought to our attention that some of the numbers in Table 3 were incorrectly reported due to data entry errors. The corrected Table 3 can be found below - modified values are marked with bold. We apologize for the inconvenience this may have caused.

**Table 3:** Swedish universities Scales of Personality (SSP) raw scores (mean [standard deviation]) and 95% confidence interval (CI) of the difference between baseline and follow-up in patients with psychotic illness and non-psychotic controls

		N	Somatic Trait Anxiety			Psychic Trait Anxiety			Stress Susceptibility		
			Baseline	Follow-up	CI	Baseline	Follow-up	CI	Baseline	Follow-up	CI
ALL	Patient	36	**2.08 (0.48)**	**2.13 (0.53)**	−0.25 – 0.15	**2.45 (0.62)**	**2.36 (0.61)**	−0.09 – 0.27	**2.46 (0.55)**	**2.47 (0.53)**	−0.19 – 0.16
	Control	76	**1.58 (0.46)**	**1.55 (0.45)**	−0.06 – 0.12	**1.66 (0.50)**	**1.48 (0.39)**	0.11–0.26	**1.87 (0.47)**	**1.82 (0.44)**	−0.05 – 0.14
ALL	Men	75	**1.68 (0.48)**	**1.71 (0.56)**	−0.14 – 0.08	**1.92 (0.68)**	**1.79 (0.66)**	0.04–0.22	**2.06 (0.61)**	**2.02 (0.58)**	−0.06 – 0.14
	Women	37	**1.85 (0.58)**	**1.78 (0.53)**	−0.08 – 0.21	**1.90 (0.60)**	**1.70 (0.55)**	0.07–0.34	**2.04 (0.46)**	**2.05 (0.53)**	−0.17 – 0.16
PATIENT	Men	28	**2.05 (0.39)**	**2.19 (0.51)**	−0.39 – 0.09	**2.47 (0.60)**	**2.43 (0.52)**	−0.17 – 0.24	**2.48 (0.60)**	**2.52 (0.46)**	−0.24 – 0.17
	Women	8	**2.21 (0.71)**	**1.93 (0.57)**	0.02–0.55	**2.38 (0.71)**	**2.09 (0.84)**	−0.17 – 0.74	**2.36 (0.38)**	**2.30 (0.76)**	−0.40 – 0.51
CONTROL	Men	47	**1.47 (0.39)**	**1.43 (0.36)**	−0.06 – 0.14	**1.60 (0.49)**	**1.41 (0.37)**	0.10–0.28	**1.81 (0.47)**	**1.72 (0.41)**	−0.02 – 0.20
	Women	29	**1.75 (0.51)**	**1.74 (0.52)**	−0.16 – 0.18	**1.77 (0.51)**	**1.59 (0.40)**	0.04–0.33	**1.96 (0.45)**	**1.98 (0.44)**	−0.21 – 0.17
		Lack of Assertiveness				Impulsiveness			Adventure Seeking		
		Baseline		Follow-up	CI	Baseline	Follow-up	CI	Baseline	Follow-up	CI
ALL	Patient	**2.50 (0.64)**		**2.43 (0.62)**	−0.13 – 0.28	2.33 (0.52)	2.25 (0.51)	−0.07 – 0.23	2.50 (0.58)	2.38 (0.61)	**−0.04 – 0.29**
	Control	**1.91 (0.40)**		**1.87 (0.43)**	−0.04 – 0.11	2.30 (0.46)	2.19 (0.47)	0.02–0.20	2.53 (0.52)	2.39 (0.51)	0.07–0.22
ALL	Men	**2.12 (0.58)**		**2.07 (0.61)**	−0.06 – 0.16	2.23 (0.47)	2.15 (0.47)	−0.01 – 0.17	2.57 (0.58)	2.42 (0.56)	0.06–0.25
	Women	**2.06 (0.54)**		**2.02 (0.44)**	− 0.07 – 0.15	2.48 (0.45)	2.34 (0.49)	0.01–0.28	2.42 (0.43)	2.33 (0.50)	− 0.01 – 0.21
PATIENT	Men	**2.55 (0.59)**		**2.48 (0.63)**	−0.18 – 0.33	2.28 (0.51)	2.21 (0.52)	−0.12 – 0.24	2.52 (0.62)	2.34 (0.65)	−0.02 – 0.38
	Women	**2.34 (0.83)**		**2.27 (0.59)**	−0.21 – 0.35	2.52 (0.51)	2.38 (0.48)	−0.22 – 0.49	2.46 (0.39)	2.54 (0.46)	−0.33 – 0.19
CONTROL	Men	**1.86 (0.40)**		**1.83 (0.45)**	−0.06 – 0.13	2.19 (0.44)	2.11 (0.44)	−0.02 – 0.20	2.61 (0.55)	2.47 (0.51)	0.04–0.24
	Women	**1.99 (0.41)**		**1.95 (0.37)**	−0.09 – 0.16	2.47 (0.44)	2.33 (0.50)	−0.00 – 0.30	2.41 (0.44)	2.27 (0.50)	0.02–0.27
		Detachment				Social Desirability			Embitterment		
		Baseline	Follow-up		CI	Baseline	Follow-up	CI	Baseline	Follow-up	CI
ALL	Patient	**2.33 (0.63)**	**2.31 (0.52)**		−0.14 – 0.17	2.77 (0.53)	2.83 (0.45)	−0.24 – 0.13	2.23 (0.50)	2.22 (0.55)	−0.15 – 0.17
	Control	**1.90 (0.43)**	**1.85 (0.43)**		−0.02 – 0.12	2.83 (0.37)	2.92 (0.35)	−0.15 - -0.03	1.60 (0.41)	1.47 (0.40)	0.05–0.20
ALL	Men	**2.16 (0.54)**	**2.13 (0.49)**		−0.05 – 0.12	2.80 (0.43)	2.88 (0.39)	−0.16 – 0.00	1.82 (0.52)	1.72 (0.59)	0.01–0.20
	Women	**1.78 (0.45)**	**1.74 (0.42)**		−0.09 – 0.18	2.82 (0.42)	2.90 (0.38)	−0.21 – 0.06	1.75 (0.56)	**1.69 (0.54)**	−0.06 – 0.18
PATIENT	Men	**2.41 (0.65)**	**2.39 (0.50)**		−0.14 – 0.20	2.82 (0.56)	2.80 (0.46)	−0.18 – 0.21	2.23 (0.44)	2.23 (0.57)	−0.20 – 0.20
	Women	**2.04 (0.52)**	**2.05 (0.53)**		−0.51 – 0.48	2.61 (0.42)	2.91 (0.44)	−0.87 – 0.26	2.23 (0.69)	2.17 (0.51)	−0.22 – 0.34
CONTROL	Men	**2.02 (0.41)**	**1.97 (0.42)**		−0.05 – 0.13	2.79 (0.34)	2.93 (0.34)	−0.21 - − 0.07	1.58 (0.39)	1.42 (0.34)	0.08–0.25
	Women	**1.71 (0.41)**	**1.65 (0.35)**		-0.07 – 0.20	2.88 (0.41)	2.90 (0.36)	−0.12 – 0.09	1.62 (0.45)	1.56 (0.47)	−0.08 – 0.20
		Trait Irritability				Mistrust					
		Baseline	Follow-up		CI	Baseline	Follow-up	CI			
ALL	Patient	2.35 (0.57)	2.13 (0.57)		0.03–0.40	2.26 (0.58)	2.26 (0.52)	− 0.13 – 0.15			
	Control	2.21 (0.48)	2.03 (0.49)		0.09–0.26	1.75 (0.50)	1.60 (0.47)	0.06–0.24			
ALL	Men	2.26 (0.49)	2.04 (0.53)		0.12–0.31	1.97 (0.56)	1.86 (0.57)	0.02–0.21			
	Women	2.24 (0.55)	2.11 (0.50)		−0.03 – 0.29	1.81 (0.59)	1.72 (0.58)	−0.03 – 0.21			
PATIENT	Men	2.36 (0.56)	2.16 (0.53)		0.01–0.38	2.28 (0.58)	2.25 (0.53)	−0.14 – 0.21			
	Women	2.32 (0.66)	2.04 (0.74)		−0.37 – 0.94	2.20 (0.62)	2.29 (0.55)	−0.24 – 0.07			
CONTROL	Men	2.20 (0.45)	1.97 (0.51)		0.11–0.34	1.79 (0.47)	1.62 (0.47)	0.05–0.28			
	Women	**2.22 (0.53)**	2.13 (0.43)		−0.05 – 0.22	1.70 (0.54)	1.56 (0.49)	−0.01 -0.29			
		Verbal Trait Aggression				Physical Trait Aggression					
		Baseline		Follow-up	CI	Baseline	Follow-up	CI			
ALL	Patient	2.01 (0.64)		2.00 (0.55)	−0.19 – 0.20	1.71 (0.57)	1.84 (0.60)	−0.31 – 0.07			
	Control	2.15 (0.47)		1.96 (0.46)	0.10–0.28	1.93 (0.57)	1.67 (0.54)	0.17–0.35			
ALL	Men	2.12 (0.53)		**2.02 (0.52)**	−0.01 – 0.21	1.92 (0.60)	1.81 (0.59)	0.00–0.23			
	Women	2.07 (0.55)		1.88 (0.43)	0.04–0.33	1.73 (0.53)	1.55 (0.46)	0.01–0.33			
PATIENT	Men	2.03 (0.62)		2.07 (0.58)	−0.26 – 0.18	1.76 (0.59)	1.86 (0.66)	−0.33 – 0.13			
	Women	1.95 (0.74)		1.79 (0.38)	−0.37 – 0.69	1.54 (0.47)	1.74 (0.39)	−0.57 – 0.16			
CONTROL	Men	2.18 (0.46)		2.00 (0.48)	0.07–0.31	2.02 (0.58)	1.78 (0.55)	0.14–0.35			
	Women	2.10 (0.50)		1.91 (0.44)	0.04–0.34	1.78 (0.54)	1.50 (0.47)	0.11–0.44			

## References

[1] Fagerberg (2018). Stability of personality traits over a five-year period in Swedish patients with schizophrenia spectrum disorder and nonpsychotic individuals: a study using the Swedish universities scales of personality. BMC Psychiatry.

